# Peripheral T-cell lymphoma presenting as an ischemic stroke in a 23-year-old woman: a case report and review of the literature

**DOI:** 10.1186/1752-1947-3-83

**Published:** 2009-10-27

**Authors:** Mariantina Fragou, Dimitrios Karakitsos, Alexandros Kalogeromitros, George Samonis, Andreas Karabinis

**Affiliations:** 1 Intensive Care Unit, General Hospital of Athens, 154 Mesogeion Avenue, Athens 11527, Greece; 2 Department of Internal Medicine, Infectious Diseases Unit, University of Crete, Crete 11244, Greece

## Abstract

**Introduction:**

Peripheral T-cell lymphoma of the unspecified variant is a highly aggressive subtype of T-cell non-Hodgkin's lymphoma. This is the first reported case of this type of lymphoma presenting as an ischemic stroke in a woman.

**Case presentation:**

A previously healthy 23-year-old woman presented with fever and hemiplegia. She was subsequently intubated after scoring 7 out of 15 at the Glasgow Coma Scale. Brain computed tomography scans of the patient depicted a massive sylvian infarction while an abdominal computed tomography scan revealed multiple enlarged abdominal lymph nodes and a retroperitoneal mass adjacent to the left psoas muscle. A diagnostic work up for inherited thrombophilia yielded negative results. Blood and cerebrospinal fluid cultures for infectious agents also gave negative results. A biopsy of the retroperitoneal mass guided by computed tomography was inconclusive. A biopsy of an enlarged inguinal lymph node of the patient, combined with an immunophenotypic analysis, revealed an unspecified variant of peripheral T-cell lymphoma. The patient underwent chemotherapy but developed multiple organ failure. She died 26 days after she was admitted to our intensive care unit.

**Conclusion:**

Peripheral T-cell lymphoma of the unspecified variant is a highly aggressive subtype of peripheral T-cell lymphomas. The latter exhibit no consistent immunophenotypic, genetic, or clinical features. Clinicians should be aware of atypical clinical presentations of the above lymphomas such as ischemic stroke.

## Introduction

Peripheral T-cell lymphoma of the unspecified variant (PTCL-U) is a highly aggressive subtype of T-cell non-Hodgkin's lymphoma. The Revised European American Lymphoma (REAL) classification cites PTCL-U as comprising a mere 6% of all surveyed cases of lymphoma, thus reflecting its rarity among the American and European populations [1,2]. Its clinical course is aggressive and may be characterized by the presence of diffuse adenopathy, extranodal disease, B-symptoms and a propensity for relapse [1-3]. We present the case of a woman who was admitted to our intensive care unit (ICU) due to cerebral infarction. Further diagnostic investigations revealed that she had PTCL-U. While a brain tumor is a main differential diagnosis in patients with ischemia, PTCL-U presenting as a cerebral infarction has not been previously described in the literature. Furthermore, we also discuss in this case report some diagnostic issues related to the causes of stroke in patients with hematological malignancies.

## Case presentation

**Figure 1:**
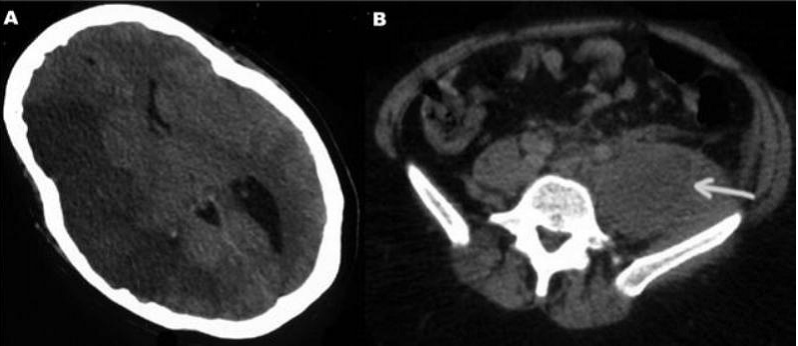
**A) Brain computed tomography scan depicting a large right-sided ischemic region and cerebral edema**. B) Abdominal computed tomography scans revealing multiple enlarged lymph nodes and a retroperitoneal mass adjacent to the left psoas muscle.

A previously healthy 23-year-old Caucasian woman presented to our emergency department with left-sided hemiplegia and a fever (39°C). She was later intubated and admitted to the ICU when she scored 7 out of 15 at the Glasgow Coma Scale. Upon admission, the patient underwent a brain computed tomography (CT) scan, which depicted a massive sylvian infarction associated with severe cerebral edema (Figure 1A); hence, she underwent a decompressive craniectomy.

Physical examination was unremarkable except for the presence of multiple palpable left-sided inguinal lymph nodes. Laboratory tests revealed leukocytosis (WBC: 17, 900 cells/mm^3^, 91% neutrophils), hemoglobin level at 10 gr/dL, elevated lactate deydrogenase at 356 IU/L and C-reactive protein at 197 and 8 mg/L.

Consequently, the patient underwent an abdominal CT scan that demonstrated multiple enlarged lymph nodes and a retroperitoneal mass adjacent to the left psoas muscle (Figure 1B). Finally, she underwent a transesophageal echocardiography that showed normal results.

Blood cultures taken from the patient were negative for bacterial, fungal and mycobacterial pathogens. Serologic tests for cytomegalovirus, herpes simplex virus, Epstein-Barr virus, Human Immunodeficiency Virus (HIV), tularaemia, *Yersinia pestis*, brucellosis, leptospirosis, Lyme disease, syphilis and Toxoplasma gondii were inconclusive. An examination of the patient's cerebrospinal fluid was negative. Results of the peripheral blood smear and bone marrow aspiration were not diagnostic.

Further laboratory tests for autoimmune disorders were also inconclusive. A diagnostic work up for inherited causes of thrombophilia such as protein C and S deficiency, antithrombin III deficiency, factor V Leiden gene mutation (associated with activated protein C resistance), prothrombin gene mutation, hyperhomocysteinemia, elevated lipoprotein (a) and polycythemia vera revealed no pathology. Furthermore, acquired prothrombotic states such as paroxysmal nocturnal hemoglobinuria, nephrotic syndrome, hyperviscosity disorders (Waldenstrom's macroglobulinemia, multiple myeloma) and sickle cell anemia were excluded conditions.

**Figure 2:**
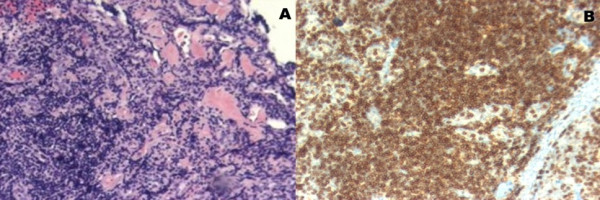
**A) Histological section showing multiple lymphocytes and zones of fibrosis consistent with an inguinal lymph node**. A hematoxylin and eosin stain magnified 100 times was used. B) Histological section of the inguinal lymph node demonstrating positive antibodies against CD3 and verifying the T-cell origin of the lymphoma.

The patient underwent a CT-guided biopsy of her retroperitoneal mass, but no specific infection and/or malignancy was identified. Finally, a biopsy of an inguinal lymph node (Figure 2A) revealed the presence of PTCL-U. An immunochemistry analysis of the patient showed positive antibodies against CD3 (Figure 2B) and CD5, thus verifying the T-cell origin of her lymphoma. Meanwhile, she showed negative antibodies against CD10, CD30, CD57 and B-cell lymphoma 6. Her CD4-to-CD8 ratio was 4:1 for T-lymphocytes. Her Ki-67 was positive for 8% to 10% of the nuclei population.

The patient underwent chemotherapy consisting of cyclophosphamide, doxorubicin, vincristine and prednisone (CHOP), but she developed multiple organ failure and died 26 days after her admission to the ICU. An autopsy revealed small periventricular and intraparenchymal mass infiltrations that caused multifocal occlusion of the small blood vessels.

## Discussion

Peripheral T-cell lymphomas comprise a heterogeneous group of tumors, which originate from mature T-cells and constitute less than 15% of all non-Hodgkin's lymphomas occurring in adults. The current World Health Organization (WHO) classification recognizes nine distinct clinicopathologic features of peripheral T-cell non-Hodgkin's lymphomas [3-5]. Viral infections such as the human T-cell leukemia virus and the Epstein-Barr virus as well as specific chromosomal translocations are implicated in the pathogenesis of PTCL-U [3]. These lymphomas, which usually affect adults at a median age of 61 years, are often associated with a poor outcome [5,6].

PTCL-U usually presents together with a generalized adenopathy and/or an extranodal disease, B-symptoms, mild anemia or thrombocytopenia, hypereosiniphilia, pruritus, and hemophagocytosis [3,6-8]. However, this type of lymphoma exhibits no consistent immunophenotypic, genetic or clinical features which makes diagnosis on a purely morphologic ground difficult. The diagnosis of PTCL-U requires careful immunophenotypic studies and can only be accurately made through exclusion [3,4]. T-cell-associated antigens such as CD3, CD5 and CD7 are variably expressed on immunohistochemistry, although one of the mature T-cell antigens (CD5 or CD7) is usually lost [9,10]. Furthermore, CD4 is more commonly expressed than CD8. This phenotypic diversity, however, does not have any obvious clinical correlation [3,4,6].

Because PTCL-U exhibits an aggressive behavior and usually presents at an advanced stage, its optimal therapy is contentious [3,4,6,11,12]. In this case, our patient presented with cerebral infarction. The differential diagnosis of stroke in young patients usually includes cardiac and hematologic diseases, inherited and acquired thrombophilias, malignancies, autoimmune disorders, inflammatory and noninflammatory vascular disorders, metabolic syndromes, and cocaine ingestion.

Meanwhile, the diagnosis of cancer leads to other possible causes of stroke such as disorders of coagulation, direct central nervous system metastases, nonbacterial thrombotic endocarditis, venous sinus occlusion, and tumor embolization [13]. Specifically, hematologic malignancies can have direct neurological effects caused by the interaction of the tumor with adjacent tissues, such as mass lesions, leptomeningeal infiltration or direct vascular occlusion. Indirect neurological effects, on the other hand, can be paraneoplastic syndromes, venous sinus occlusions and disorders of coagulation [14].

An autopsy of the patient revealed small periventricular and intraparenchymal mass infiltrations that caused small blood vessel occlusion. However, a brain CT scan upon her admission revealed a massive sylvian infarction. It should be noted that that no follow-up brain Magnetic Resonance Imaging (MRI) was performed; hence no definite pathophysiologic mechanism could be suggested to explain the autopsy results. The vessel obstruction could be attributed either to a dissemination of the lymphoma cells or to other paraneoplastic phenomena.

## Conclusion

This case illustrates that peripheral T-cell lymphomas of the unspecified variant exhibit an aggressive clinical course and are usually associated with a poor outcome. Their clinical and pathologic characteristics are not consistent, which make diagnosis difficult. Clinicians should thus be conscious of ischemic stroke and other atypical clinical presentations of this type of lymphoma.

## Abbreviations

REAL classification: Revised European American Lymphoma classification; WBC: white blood cells; WHO: World Health Organization; ICU: Intensive Care Unit; CNS: central nervous system; PTCL-U: peripheral T-cell lymphoma of the unspecified variant; CHOP: cyclophosphamide, doxorubicin, vincristine, prednisone; CT: computed tomography; HIV: Human Immunodeficiency Virus; MRI: Magnetic Resonance Imaging.

## Consent

Written informed consent was obtained from the patient's next-of-kin for the publication of this case report and any accompanying images. A copy of the written consent is available for review by the Editor-in-Chief of this journal.

## Competing interests

The authors declare that they have no competing interests.

## Authors' contributions

MF collected the data and drafted the manuscript. DK, AK, GS and KA participated in all medical interventions and drafted the final version of this manuscript. All authors read and approved the final manuscript.

## References

[B1] Armitage J Weisenburger D New approach to classifying Non-Hodgkin's lymphomas: Clinical features of the major histologic subtypes J Clin Oncol 1998 16 2780 95 9704731 10.1200/JCO.1998.16.8.2780

[B2] A clinical evaluation of the International Lymphoma Study Group classificationof non-Hodgkin's lymphoma. The non-Hodgkin's lymphoma classification project Blood 1997 89 3909 18 9166827

[B3] Rodriguez-Abreu D Filho VB Zucca E Peripheral T-cell lymphomas, unspecified (or not otherwise specified): a review Hematol Oncol 2008 26 8 20 10.1002/hon.836 18050364

[B4] Jaffe ES Ralfkiaer E Jaffe ES, Harris NL, Stein H, Vardiman JW Mature T-cell and NK-cell neoplasms: introduction World Health Organization classification of tumours: Pathology and genetics of tumours of haematopoietic and lymphoid tissues 2001 3 Lyon: IARC Press 191 194

[B5] Rizvi MA Evens AM Tallman MS Nelson BP Rosen ST T-cell non-Hodgkin lymphoma Blood 2006 107 1255 1264 10.1182/blood-2005-03-1306 16210342

[B6] Rüdiger T Weisenburger DD Anderson JR Armitage JO Diebold J MacLennan KA Nathwani BN Ullrich F Müller-Hermelink HK Peripheral T-cell lymphoma (excluding anaplastic large-cell lymphoma): results from the Non-Hodgkin's Lymphoma Classification Project Ann Oncol 2002 13 140 149 10.1093/annonc/mdf033 11863096

[B7] López-Guillermo A Cid J Salar A López A Montalbán C Castrillo JM González M Ribera JM Brunet S García-Conde J Fernández de Sevilla A Bosch F Montserrat E Peripheral T-cell lymphomas: initial features, natural history, and prognostic factors in a series of 174 patients diagnosed according to the REAL classification Ann Oncol 1998 9 8 849 855 10.1023/A:1008418727472 9789607

[B8] Kojima H Hasegawa Y Suzukawa K Mukai HY Kaneko S Kobayashi T Kamoshita M Shinagawa A Komeno T Komatsu T Mitsuhashi S Kawachi Y Yamashita Y Mori N Nagasawa T Clinicopathological features and prognostic factors of Japanese patients with "peripheral T-cell lymphoma, unspecified" diagnosed according to the WHO classification Leuk Res 2004 28 12 1287 1292 10.1016/j.leukres.2004.04.016 15475070

[B9] Weiss LM Crabtree GS Rouse RV Warnke RA Morphologic and immunologic characterization of 50 peripheral T-cell lymphomas Am J Pathol 1985 118 2 316 324 3155915 PMC1887884

[B10] Borowitz MJ Reichert TA Brynes RK Cousar JB Whitcomb CC Collins RD Crissman JD Byrne GE Jr The phenotypic diversity of peripheral T-cell lymphomas: the Southeastern Cancer Study Group experience Hum Pathol 1986 17 6 567 574 10.1016/S0046-8177(86)80128-9 3011639

[B11] Armitage JO Vose JM Weisenburger DD Towards understanding the peripheral T-cell lymphomas Ann Oncol 2004 15 10 1447 1449 10.1093/annonc/mdh409 15367401

[B12] Gallamini A Stelitano C Calvi R Bellei M Mattei D Vitolo U Morabito F Martelli M Brusamolino E Iannitto E Zaja F Cortelazzo S Rigacci L Devizzi L Todeschini G Santini G Brugiatelli M Federico M Intergruppo Italiano Linfomi Peripheral T-cell lymphoma unspecified (PTCL-U): a new prognostic model from a retrospective multicentric clinical study Blood 2004 103 2474 10.1182/blood-2003-09-3080 14645001

[B13] Rogers LR Cerebrovascular complications in patients with cancer Semin Neurol 2004 24 4 453 460 10.1055/s-2004-861539 15637656

[B14] Glass J Neurologic complications of lymphoma and leukemia Semin Oncol 2006 33 3 342 347 10.1053/j.seminoncol.2006.03.004 16769423

